# Coexistence of Schizophrenia, Epilepsy, and Polyglandular Autoimmune Syndrome

**DOI:** 10.5152/eurasianjmed.2023.22251

**Published:** 2023-06-01

**Authors:** Halil Özcan

**Affiliations:** Department of Psychiatry, Atatürk University, School of Medicine, Erzurum, Turkey

Dear editor,

Polyglandular autoimmune syndrome (PAS) is a group of disorders identified by the association of endocrine and nonendocrine organ involvement. There are 4 subtypes. Type I is characterized by comorbidities of hypoparathyroidism, Addison disease, and mucocutaneous candidiasis. Type II; Addison disease, insulin-dependent diabetes mellitus, and/or autoimmune thyroid disease. Type III; insulin-dependent diabetes mellitus and autoimmune thyroid disease without Addison disease.^1^ Type IV is a combination of endocrine and autoimmune diseases including insulin-dependent diabetes mellitus, pernicious anemia, alopecia, vitiligo or neuromuscular junction disorder, thyroid disease, or hypoparathyroidism without Addison disease.^2^ There are case reports representing comorbidities of PAS and neuropsychiatric disorders such as anorexia nervosa, paranoid schizophrenia, obsessive-compulsive disorder, narcolepsy, Munchausen syndrome, myopathy, dystonia, etc.^2-7^ According to our knowledge, this is the first case with type IV PAS having type I insulin-dependent diabetes mellitus, pernicious anemia, and generalized vitiligo with the coexistence of schizophrenia and epilepsy.

Twenty-four-year-old male college student, with previous diagnoses of type I diabetes mellitus, pernicious anemia, and generalized vitiligo had been hospitalized at the internal medicine inpatient department due to unregulated blood glucose levels. During clinical follow-up, he had been consulted with the psychiatry department because of noncompliance to treatment regimens, irritability, and aggressive behaviors toward other people. In psychiatric anamnesis, his parents declared that for 2 years, they had noticed some odd behaviors in their son, such as talking to himself, suspiciousness of others, aggressiveness, social isolation, etc. Also, he had been noncompliant with his insulin treatment during this 2-year period. His psychiatric symptoms have been continuing during this 2-year period. On admission to the psychiatric unit of another hospital with the insistence of his parents to admit 3 months before, he had been diagnosed with schizophrenia and an atypical antipsychotic (aripiprazole 15 mg/day) and a benzodiazepine (lorazepam 2 mg/day) had started. He had been discharged on the fourth day of hospitalization due to his inconvenient behaviors such as verbal and physical assault on clinical staff and damaging the hospital equipment. After 10 days of usage, he stopped taking aripiprazole and lorazepam without medical advice as his psychotic symptoms were partially alleviated. Three months later, he was rehospitalized to our inpatient clinic because of the exacerbation of psychotic symptoms. On physical examination, generalized vitiligo (nearly involving 70% of his full body skin) was detected. His mental state examination revealed that he was fully conscious, his appearance was compatible with his socio-cultural level, his speech was clear, and his orientation to time, place, and person was intact. Immediate, recent, and remote memory was normal. His affect was irritable, and his mood was euthymic. No pathology was defined in thought structure and process. In thought content, paranoid and persecutory delusions, and in perception examination, possible visual hallucinations were detected. Aggressive behaviors toward other people and odd behaviors such as talking to himself were seen. His family history of psychiatric disorders was unremarkable. On laboratory examinations, his blood glucose levels were generally found as higher than normal (varying between 300 mg/dL and 850 mg/dL, normal ranges 70-100 mg/dL). His serum vitamin B12 level was 112 pg/mL (normal ranges 200-900 pg/mL). The mean corpuscular volume of red blood cells was 122 femtoliters (fL) (normal ranges 80-100 fL). Folic acid level, thyroid, parathyroid, kidney and liver functions, and serum electrolyte levels except sodium were within normal ranges. Sodium level was 123 (normal ranges 135-147 milliequivalents), and it was thought as related to the high blood glucose level. Aripiprazole 15 mg/day was started due to a diagnosis of schizophrenia. Three days later, he was transferred to the internal medicine inpatient clinic due to the occurrence of diabetic ketoacidosis. Five days later, a significant decrease in psychotic symptoms was observed and he was transferred to the psychiatry inpatient clinic. During hospitalization in our inpatient clinic, his mother mentioned about the seizures that the patient has for the first time, as contractions starting on the right hand and spreading to all extremities causing him to fall and leading to loss of consciousness. These seizures started nearly 4 years ago, at first they had been occurring once in a few months but nowadays they had been occurring more often nearly 3 times a month. One of his aunts and cousins had been on an antiepileptic treatment due to a diagnosis of epilepsy. His neurologic examination was unremarkable. His brain magnetic resonance imaging showed no pathology. Electroencephalography revealed sharp waves on the left frontal area. He was diagnosed with complex partial epilepsy and levetiracetam 1000 mg/day was started. A brief psychiatric rating scale (BPRS) was applied for identifying and measuring the severity of psychotic symptoms. The BPRS score on the first day of hospitalization was 71 and on his 15th day of hospitalization at discharge from the psychiatry inpatient clinic dropped to 21 points. Clinical follow-up was recommended. On his mental and neurologic examination on the third month of discharge, no psychotic symptoms and/or seizures have been observed and/or declared by him and/or his parents. His blood glucose levels were generally measured in normal ranges. Also, his mother declared that he had never been better during these 2 years, and his functionality significantly increased (achieved success in school, had social activities with his friends, etc.). We think that successful treatment of schizophrenia and epilepsy increased his adherence to diabetes mellitus treatment.

Cases with PAS type II and schizophrenia, a well-known psychotic disorder, exist in the literature.^5^ A genetic relationship between PAS, progressive myoclonic epilepsy, and holoprosencephaly-1 is reported.^8^ It is also known that there is a genetic link between holoprosencephaly and schizophrenia.^9^ In our case, the patient was diagnosed with PAS type IV and secondary generalized epilepsy. There may be a common genetic background between type 1 diabetes mellitus and epilepsy, also hypoglycemia and hyperglycemia may cause epileptic seizures.^10^ We excluded the effects of blood glucose dysregulation on seizures with the data of his blood glucose levels which were measured by his parents as in normal ranges or mildly high following the seizures. Epilepsy, especially temporal lobe epilepsy, and postictal or interictal psychosis may co-occur in autoimmune diseases.^11^ Studies have demonstrated the presence of antibodies directed against synaptic autoantigens in approximately 10% of cases of sporadic epilepsy.^12^ These antibodies may be associated with a purely psychiatric phenotype. Patients with postictal psychosis show higher levels of synaptic autoantibodies in serum than patients with epilepsy without psychosis.^12^ Also vitamin B12 deficiency has been related to many neuropsychiatric symptoms such as psychosis, mania, depression, cognitive impairment, delirium, etc.^13,14^ In our patient, psychotic symptoms alone may be related to schizophrenia diagnosis on its own, because psychotic symptoms alleviated in a short time with an antipsychotic regimen before antiepileptic and vitamin B12 treatments, and also might be associated or augmented with epilepsy and/or vitamin B12 deficiency. In addition in a scientific paper, PAS is disguised as a mental illness.^15^ We also think that PAS, psychosis and/or schizophrenia, epilepsy, vitiligo, and pernicious anemia may all be a part of genetic autoimmune syndrome. In this case, we were unable to make a genetic analysis. We think further research on this topic may lead to new biological perspectives on the biology of schizophrenia as a psychotic disorder, epilepsy, and PAS.
